# Differences in Rat Dorsal Striatal NMDA and AMPA Receptors following Acute and Repeated Cocaine-Induced Locomotor Activation

**DOI:** 10.1371/journal.pone.0037673

**Published:** 2012-05-24

**Authors:** Dorothy J. Yamamoto, Nancy R. Zahniser

**Affiliations:** 1 Department of Pharmacology, School of Medicine, University of Colorado Anschutz Medical Campus, Aurora, Colorado, United States of America; 2 Neuroscience Program, School of Medicine, University of Colorado Anschutz Medical Campus, Aurora, Colorado, United States of America; INSERM/CNRS, France

## Abstract

Sprague-Dawley rats can be classified as low or high cocaine responders (LCRs or HCRs, respectively) based on their locomotor activity induced by an acute low dose of cocaine. Upon repeated cocaine exposure, LCRs display greater locomotor sensitization, reward, and reinforcement than HCRs. Altered glutamate receptor expression in the brain reward pathway has been linked to locomotor sensitization and addiction. To determine if such changes contribute to the differential development of locomotor sensitization, we examined protein levels of total, phosphorylated, and cell surface glutamate N-methyl D-aspartate (NMDA) and α-amino-3-hydroxy-5-methylisoxazole-4-propionate (AMPA) receptors (Rs) following acute or repeated cocaine (10 mg/kg, i.p.) in LCRs, HCRs and saline controls. Three areas involved in the development and expression of locomotor sensitization were investigated: the ventral tegmental area (VTA), nucleus accumbens (NAc) and dorsal striatum (dSTR). Our results revealed differences only in the dSTR, where we found that after acute cocaine, GluN2B^Tyr-1472^ phosphorylation was significantly greater in LCRs, compared to HCRs and controls. Additionally in dSTR, after repeated cocaine, we observed significant increases in total GluA1, phosphorylated GluA1^Ser-845^, and cell surface GluA1 in all cocaine-treated animals vs. controls. The acute cocaine-induced increases in NMDARs in dSTR of LCRs may help to explain the more ready development of locomotor sensitization and susceptibility to addiction-like behaviors in rats that initially exhibit little or no cocaine-induced activation, whereas the AMPAR increases after repeated cocaine may relate to recruitment of more dorsal striatal circuits and maintenance of the marked cocaine-induced locomotor activation observed in all of the rats.

## Introduction

Initial responsiveness to cocaine and susceptibility to cocaine addiction varies among individuals [1], [2]. Studies using animal models have shown that the progression from initial drug use to addiction involves synaptic plasticity in brain pathways involved in reward and reinforcement, with the brain neurotransmitter glutamate being essential for this plasticity [3], [4], [5]. Better understanding of how individuals differ in cocaine-induced glutamate neuroplasticity may help to explain why individuals vary in their susceptibility to addiction. Further, this understanding may identify new molecular targets that block and/or reverse drug-induced changes in synaptic plasticity, which would be potentially useful for preventing relapse and/or treating drug addiction.

A number of animal models have been utilized to study individual differences in susceptibility to cocaine. For example, anxiety response to novel environment, impulsivity, and locomotor activity in a novel environment [6], [7], [8], [9] are models used to predict later cocaine-induced addiction-like behaviors. Our model differs in that we have used initial drug response to classify adult outbred male Sprague-Dawley rats as either low or high cocaine responders (LCRs or HCRs, respectively). Specifically, the LCR/HCR classification is based on the median split of the group locomotor activity in the first 30 min following an acute, relatively low dose of cocaine [(10 mg/kg.], [ i.p.) 10], [11,12]. At this dose, LCRs consistently develop locomotor sensitization in response to repeated cocaine whereas HCRs do not. Despite the differential drug locomotor response, however, LCRs and HCRs display similar levels of locomotor activity in the novel environment before cocaine injection and similar brain cocaine levels after injection [12]. LCRs also exhibit increased sensitivity to the discriminative stimulus properties of low dose cocaine and develop conditioned place preference and motivation to self-administer cocaine more readily than HCRs, suggesting a greater vulnerability of LCRs to the rewarding and reinforcing effects of cocaine [13], [14], [15]. Dopamine (DA) clearly plays a role initially in these individual differences because the first dose of cocaine inhibits DA transporter (DAT)-mediated clearance of DA and increases extracellular DA in the nucleus accumbens (NAc) and dorsal striatum (dSTR) to a lesser extent in LCRs than HCRs [10], [16]. However, with repeated cocaine, these differences disappear, in parallel with development of locomotor sensitization to cocaine in LCRs [11], [16].

Ionotropic glutamate receptor-mediated signaling is known to play a critical role in locomotor sensitization because inhibition of NMDA or AMPA receptors (NMDARs and AMPARs) prevents the development and/or expression of locomotor sensitization [17]. The neuroplastic changes associated with development of behavioral sensitization are thought to contribute to increased incentive salience of the drug and the drug craving associated with addiction [18]. Interestingly, repeated cocaine exposure increases expression of NMDAR and AMPAR subunits in the VTA and NAc only in rats that develop locomotor sensitization [19], [20]. Similarly, after withdrawal from repeated treatment, increased cell surface expression of AMPARs GluA1 and GluA2 in the NAc occurs only in sensitized rats [21].

Phosphorylation of specific NMDAR and AMPAR subunits is also important for receptor activity and neuroplasticity. Acute cocaine increases striatal NMDAR GluN2B^Tyr-1472^ and AMPAR GluA1^Ser-845^ phosphorylation levels in juvenile mice [22] and decreases NAc GluN2B^Ser-1303^ phosphorylation in adult rats [23], whereas enhanced GluA1^Ser-831^ phosphorylation in rat dSTR is observed at 20 and 40 minutes after repeated cocaine [24]. Here, we asked whether glutamate receptor expression is associated with differential LCR/HCR cocaine-induced initial locomotor activity and/or sensitization by measuring total and cell surface expression, as well as phosphorylation, of NMDAR and AMPAR subunits in relevant brain regions of rats classified as LCRs or HCRs at several times after cocaine exposure. We also investigated overall low dose cocaine-induced glutamate receptor differences by comparing results in the cocaine-treated rats vs. saline controls. We found acute cocaine-induced increases in phosphorylated GluN2B^Tyr-1472^ (pGluN2B^Tyr-1472^) in LCRs that are consistent with the preferential development and expression of locomotor sensitization in LCRs, as compared to HCRs. We found increases in both total and cell surface GluA1 and in pGluA1^Ser-845^ in dSTR of all rats treated repeatedly with cocaine. These changes may increase the sensitivity of the neurons in the dSTR, which could help to maintain the high levels of cocaine-induced locomotor activation observed in all of the rats after repeated cocaine exposure.

## Materials and Methods

### Ethics Statement

All procedures were approved by the University of Colorado Denver IACUC. This research program operates in accordance with the National Institutes of Health's guidelines (NIH Publication No. 80-23, revised 1996).

### Animals

A total of 110 male outbred Sprague-Dawley rats (Charles Rivers Laboratories, Wilmington, MA) weighing 160–180 g on arrival were used. They were housed in groups of 4 with food and water available *ad libitum*. A 12-hr light-dark cycle was used. Rats were acclimated to the colony room for 1 week before experiments commenced.

### Experimental groups

Five experimental groups were used (Table 1). In acute group 1 (n = 24) we measured total and phosphorylated NMDAR and AMPAR subunits at 40 min after acute cocaine or saline. In acute group 2 (n = 18) we measured total subunits 24 hr after acute cocaine or saline, and in repeated group 3 (n = 20) we measured total and phosphorylated subunits 40 min after day 7 of repeated cocaine or saline. In acute group 4 (n = 24) we measured cell surface receptors 40 min after acute cocaine or saline, and in repeated group 5 (n = 24) we measured cell surface receptors 40 min after day 7 of repeated cocaine or saline. (−)Cocaine hydrochloride was administered at 10 mg/kg, i.p. [gift from the National Institute on Drug Abuse (RTI International, Research Triangle Park, NC)], and saline at 1 mg/mL, i.p.

**Table 1:** Summary of five experimental groups and their 30 min post-injection cocaine-induced locomotor activity.

Group	Cocaine (n)	Saline (n)	Group	Day 1 activity*	Day 7 activity*	Day 1 activity**
1. Acute	16	8	LCR	347±102	NA	4.1
40 min			HCR	1410±179	NA	
2. Acute	14	4	LCR	1170±184	NA	2.2
24 hr			HCR	2570±217	NA	
3. Repeated	16	4	LCR	871±247	2750±648	3.3
40 min			HCR	2860±225	3700±685	
4. Acute	18	6	LCR	331±57	NA	3.2
40 min			HCR	1050±156	NA	
5. Repeated	18	6	LCR	432±91	2800±461	3.8
40 min			HCR	1660±192	2530±579	

* Mean ± SEM, NA = not applicable,

** = Ratio of HCR/LCR.

### Locomotor activity

Locomotor activity was measured during the light cycle. All five groups were habituated first for 60 min to the behavioral testing room and then for 90 min to the open field activity chambers (San Diego Instruments, San Diego, CA). At 90 min rats were removed, injected with either cocaine or saline, and returned to the chambers for 30 min. Locomotor activity was recorded throughout the 120-min period in the activity chambers as consecutive horizontal photobeam breaks, converted to distance traveled (cm) per 10 min. The group median of the distance traveled in the 30 min after cocaine injection was used to divide each respective group into LCRs and HCRs. Rats in the acute groups were sacrificed 40 min (groups 1 and 4) or 24 hr (group 2) after injection; and their brains were rapidly removed, chilled, dissected and prepared for Western blotting or cell surface receptor assays. For locomotor sensitization, repeated groups 3 and 5 received injections of cocaine or saline once daily for six additional days. Locomotor activity was measured on days 1, 3, 5, and 7. On day 7 rats were sacrificed 40 min after injection, and their brain tissue was prepared in a manner identical to the acute groups.

### Western blotting

To measure subunits of NMDARs (GluN1, GluN2A, GluN2B) and AMPARs (GluA1, GluA2) and phosphorylation of GluN2B and GluA1 with semi-quantitative western blotting, the ventral tegmental area (VTA), NAc, and dSTR were quickly dissected from chilled brain slices (Vibratome, Ted Pella, Inc., Redding, CA) and sonicated in ice-cold 1× STE (1% SDS, 10 mM Tris, 1 mM EDTA, pH 8.0) with protease and phosphatase inhibitors (1 µg/mL aprotinin, 1 µg/mL leupeptin, 0.1 mM 4-(2-Aminoethyl) benzenesulfonyl fluoride hydrochloride,1 mM NaF, 0.1 mM Na orthovanadate). Total protein concentrations were determined by bicinchoninic acid (BCA) protein assay (Fisher, Rockford, IL), and samples were stored at −80°C. Samples (8–20 µg protein/lysate; confirmed in preliminary experiments to be in the linear response range of each primary antibody) were loaded and electrophoresed under reducing conditions on 7.5% polyacrylamide gels, transferred to Polyscreen PVDF transfer membranes, blocked with 5% non-fat dry milk or BSA in Tris-buffered saline (140 mM NaCl, 20 mM Tris pH 7.6) with 0.1% Tween 20 (TTBS) for 1 hr and then probed with primary antibody overnight at 4°C. Primary antibodies used were from Millipore (Danvers, MA): GluA1 (1∶1000), GluA2 (1∶2000), pGluN2B^Ser-1303^ (1∶500); Phosphosolutions (Aurora, CO): GluN1 (1∶2000), GluN2A (1∶1000), GluN2B (1∶3000), pGluA1^Ser-845^ (1∶1000), pGluA1^Ser-831^ (1∶500), pGluN2B^Tyr-1472^ (1∶1,500); Cell Signaling Technology (Danvers, MA): β-actin (1∶1000). Blots were washed in TTBS, incubated with HRP-conjugated secondary antibody (BioRad, Hercules, CA) for 1 hr at room temperature and rinsed in TTBS. Chemiluminescent substrate was used for immunodetection.

### Cell surface receptor assay

Our cell surface receptor assay was adapted from Boudreau and Wolf [25]. Chilled brains were sliced (400 µm) at the level of the NAc and dSTR. Mini-slices of the NAc or dSTR were dissected out in ice-cold artificial cerebral spinal fluid (aCSF:145 mM NaCl, 2.7 mM KCl, 1.2 mM CaCl_2_, 1 mM MgCl_2_, 2 mM NaH_2_PO_4_, pH 7.4). Half of the slices were incubated in ice-cold aCSF spiked with 2 mM BS^3^ (Bis [sulfosuccinimidyl]suberate; Fisher) with gentle agitation for 30 min at 4°C. Cross-linking was terminated by quenching with 100 mM glycine for 10 min at 4°C. Following brief centrifugation, the supernatant was discarded. The washed slices were resuspended in ice-cold lysis buffer containing protease and phosphatase inhibitors, as described in the previous section, and homogenized by a 5-s sonication. The other half of the slices were suspended in the same ice-cold lysis buffer immediately upon dissection and sonicated. Lysate protein concentrations were determined by the BCA assay. Samples were stored at −80°C until analysis by western blotting, as described above, with the exception that 6% polyacrylamide gels were used.

### Data analysis

Group data are expressed as mean values ± SEM, and significance was set at p<0.05. Sensitization of cocaine-induced locomotor activity was defined for both individual rats and LCR/HCR groups as day 7 activity ≥2× day 1 activity. Two-way analysis of variance (ANOVA; GraphPad Software, La Jolla, CA) with time as the repeated measure, and Bonferroni post hoc tests were used to compare locomotor activity of LCRs, HCRs, and saline-treated rats on treatment days 1, 3, 5, and 7.

For semi-quantitative analysis of western blots, images were captured using the Chemiimager 4400 low light imaging system (Alpha Innotech; Cell Biosciences, Inc., Santa Clara, CA), and band densities were measured using AlphaEase™ and Excel software. Expression levels of total, phosphorylated, and cell surface receptor subunits were normalized to β-actin or α-tubulin to account for differences in sample loading. A ratio of phosphorylated to total receptor subunit was used to assess cocaine-induced differences in phosphorylation. For measurements of cell surface receptors, values for total – intracellular (surface), intracellular, and total protein per lane in cocaine-injected rats were normalized to saline-injected control values. Samples were run in duplicate, and results were averaged. One-way ANOVAs with Bonferroni post hoc tests were used to determine significant differences between groups for both total and phosphorylated glutamate receptor subunit expression and cell surface GluN2B, GluA1, and GluA2 expression. Student's t-tests were used to determine significant differences between cocaine and saline groups. Two statistical outliers were removed from the analysis of pGluN2B^Tyr-1472^ levels. In the repeated cocaine dSTR cell surface study one rat died, one sample was lost, and one statistical outlier was removed.

Pearson r values (GraphPad Software) were used to compare overall relationships between individual rats' cocaine-induced activity and glutamate receptor parameters.

## Results

**Figure 1:**
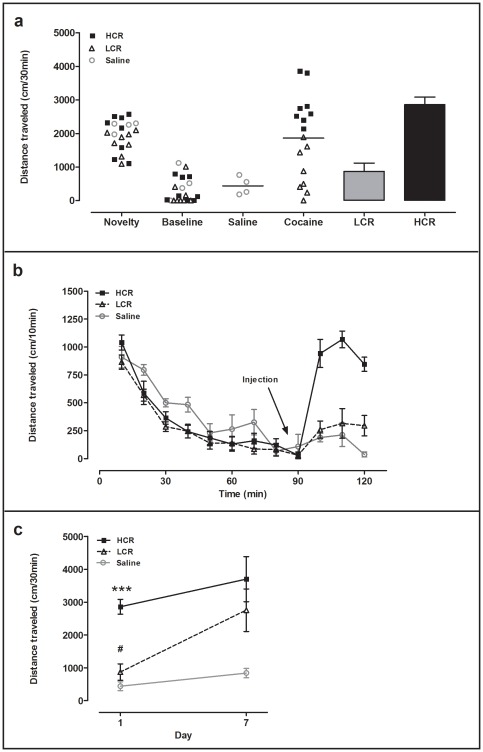
Representative results for group 3 showing LCR/HCR classification and development of locomotor sensitization in LCRs. (**a**) LCR/HCR classification is based on initial cocaine-induced open field locomotor activity on day 1. Novelty (first 30 min), baseline (30 min before injection), and 30 min post-injection activity is shown for group 3 (see Table 1) saline control rats (n = 4; horizontal line = median) and cocaine-treated (n = 16; 10 mg/kg, i.p.; horizontal line = median) rats. The subsequent classification of the cocaine-treated rats as either LCRs (n = 8; white bar; mean values ± SEM) or HCRs (n = 8; black bar) was based on the group median split. (**b**) Locomotor activity is shown over the 90 min pre- and 30 min post-injection (arrow) period for saline controls, LCRs, and HCRs on day 1. (**c**) Cocaine-induced locomotor activity on days 1 and 7 of the seven-day once-daily treatment illustrates development of locomotor sensitization only in the LCRs (***p<0.0001 LCR vs. HCR, day 1; ^#^p<0.05 LCR day 1 vs. LCR day 7).

Experiments were conducted with five separate groups of rats (Table 1). Fig. 1 shows results for group 3 as representative for LCR/HCR classification and locomotor sensitization. Rats were classified as LCRs or HCRs based on the median split of each group's cocaine-induced locomotor activity in the 30 min after an acute 10 mg/kg (i.p.) cocaine injection (Fig. 1a). Locomotor activity over the entire 120-min behavioral recording period (90 min before injection and 30 min after injection) is shown for this group of LCRs, HCRs and saline controls in Fig. 1b. Prior to cocaine injection, LCRs, HCRs and saline controls did not differ in either novelty-induced (0–30 min) or baseline (60–90 min) activity; however, after injection, HCRs became markedly more active than either LCRs or controls (Figs. 1a–b). In all five experimental groups, HCRs exhibited 2- to 4-fold higher acute cocaine induced-locomotor activity than LCRs (see Table 1). To induce locomotor sensitization, rats in the two repeated treatment groups were given 6 additional once-daily injections of cocaine (LCRs and HCRs) or saline (controls).The first 30 min of cocaine-induced locomotor activity on days 7 and 1 were compared to determine sensitization (see Methods: *Data analysis*; Table 1). The representative results from group 3 are shown in Fig. 1c. On day 1, there was a significant LCR/HCR difference, but no LCR/saline difference. However, over the 7-day cocaine regimen, the LCR activity increased and eventually was comparable to that of the HCRs, which did not change over the 7 days. Post-hoc tests on day 7 revealed a significant ≥2-fold difference (p<0.05) between locomotor activity of the LCR and saline groups, but not LCR and HCR groups, confirming the development of sensitization in LCRs over the 7-day repeated cocaine treatment.

For the first three groups of rats, the protein levels of NMDAR subunits (GluN1, GluN2A, and GluN2B) and AMPAR subunits (GluA1and GluA2) in three brain areas – the VTA, NAc, and dSTR – were compared by immunoblotting between LCRs, HCRs, all cocaine-treated rats and saline controls. Three time points after treatment were examined: 40 min after acute injection of cocaine or saline (group 1), 24 hr after acute injection of cocaine or saline (group 2), and 40 min after injection on day 7 of repeated cocaine or saline (group 3). No differences were found in these experiments.

**Figure 2:**
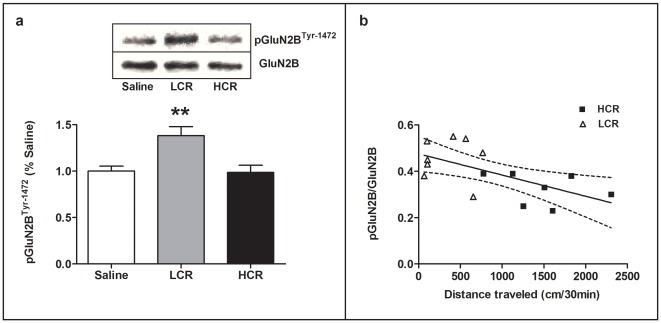
Increased striatal GluN2B^Tyr-1472^ phosphorylation in LCRs after acute cocaine. (**a**) The level of NMDAR pGluN2B ^Tyr-1472^ was selectively increased in dSTR of LCRs, compared to HCRs and saline controls, at 40 min after an acute 10 mg/kg i.p. cocaine injection (group 1, see Table 1). Representative western blots are shown in the panel above the bars. Mean values ±SEM for the ratios of pGluN2B^Tyr-1472^ to total GluN2B in dSTR are shown for each treatment. Saline: n = 7, LCR: n = 8, HCR: n = 7. ** p<0.01: LCR vs. HCR, LCR vs. Saline. One Saline and one HCR were removed from the analysis (see Methods, Data Analysis). (**b**) For all of the cocaine-treated rats, there was a significant negative correlation between cocaine-induced locomotor activity and pGluN2B^Tyr-1472^ levels in the dSTR; r^2^ = 0.3905, p<0.05; n = 15. LCR = open triangle, HCR = closed square. One HCR was removed from the analysis (see Methods, Data Analysis).

To determine whether glutamate receptor phosphorylation states differed after cocaine treatment, we measured levels of phosphorylation in GluN2B, (Ser-1303 and Tyr-1472) and GluA1 (Ser-831 and Ser-845) in the VTA, NAc and dSTR. We compared these levels in LCRs, HCRs, all cocaine-treated rats and saline controls in both acute (group 1; 40 min after acute cocaine or saline) and repeated (group 3; 40 min after day 7 of repeated cocaine or saline) treatment groups. In acutely treated rats, one-way ANOVA revealed significantly different levels only of pGluN2B^Tyr-1472^ in the dSTR [*F*(2,19) = 8.375, p<0.01; Fig. 2a]. Bonferroni post-hoc tests revealed that pGluN2B^Tyr-1472^ in LCRs was increased by 40% (p<0.01) and 38% (p<0.01) as compared to HCRs and controls, respectively. Further, we compared all of the acutely cocaine-treated rats in group 1, regardless of LCR/HCR classification, and found a significant negative correlation between cocaine-induced locomotor activity (30 min post-injection) and the phosphorylation state of GluN2B^Tyr-1472^ in the dSTR, [r^2^ = 0.3905, p<0.05; Fig. 2b]. We also observed a trend for a similar negative correlation between cocaine-induced locomotor activity and the phosphorylation state of GluN2B^Tyr-1472^ in the NAc (r^2^ = 0.20, p = 0.08; data not shown).

**Figure 3:**
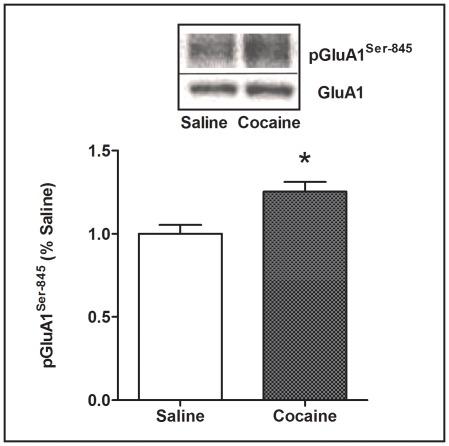
Increased striatal GluA1^Ser-845^ phosphorylation after repeated low dose cocaine. The level of pGluA1^Ser-845^ was increased in the dSTR of cocaine-treated rats vs. saline controls 40 min after day 7 of repeated once-daily 10 mg/kg i.p. cocaine or saline injections (group 3, see Table 1). Representative western blots are shown in the panel above the bars for the ratios of pGluA1^Ser-845^ to total GluA1 in dSTR. Mean values ± SEM are shown for Saline: n = 4, Cocaine: n = 16. *p<0.05, Cocaine vs. Saline.

Following repeated cocaine or saline treatment, ANOVA revealed no significant group differences between LCRs, HCRs, and controls in any of the phosphorylated NMDAR or AMPAR subunits in any of the brain regions measured. However, for the repeated treatment groups, a t-test revealed that AMPAR pGluA1^Ser-845^ increased by 25% (p<0.05) in dSTR in the cocaine vs. saline control groups (Fig. 3).

**Figure 4:**
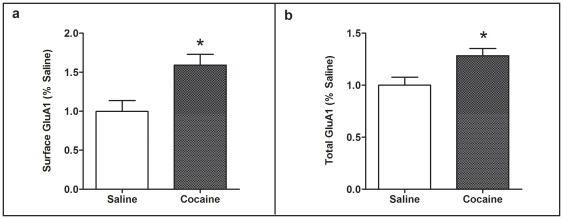
Increased striatal cell surface and total GluA1 after repeated low dose cocaine. (**a**) Cell surface GluA1, as determined by Total-Intracellular levels, was increased in dSTR of cocaine-treated rats vs. saline controls 40 min after day 7 of repeated once-daily 10 mg/kg i.p. cocaine or saline injections (group 5, see Table 1). (**b**) Total GluA1 in dSTR was increased in cocaine-treated rats vs. saline controls. Mean values ± SEM are shown for Saline: n = 6, Cocaine: n = 15). *p<0.05, Cocaine vs. Saline. Three cocaine rats were removed from analysis (see Methods, Data Analysis).

Previous studies have found changes in GluA1, GluA2, and GluN2B cell-surface levels in NAc after repeated cocaine [21], [25], [26]. To determine whether cell surface expression of NMDAR GluN2B and AMPAR GluA1 and GluA2 subunits differed after cocaine treatment we indirectly measured total and intracellular subunit levels in NAc and dSTR minislices using BS^3^ in acutely (group 4 sacrificed 40 min after acute cocaine or saline) and repeatedly (group 5 sacrificed 40 min after day 7 of repeated treatment) treated groups. No significant differences were observed in total, intracellular, or cell surface GluN2B or GluA2 subunits in either brain region of the LCRs, HCRs or saline controls in either treatment group (data not shown). ANOVA revealed no group differences (LCR, HCR, saline) for the cell surface (p = 0.12), but a strong trend for differences in total GluA1 in dSTR (p = 0.06). However, although no differences in total GluA1 were seen in our initial experiments (group 3), with a larger n for controls (group 5) and possible experimental differences, t-tests revealed a significant (p<0.05) increase in both cell surface (Fig. 4a) and total (Fig. 4b) GluA1 in dSTR of the cocaine vs. saline treatment groups.

## Discussion

Here, we investigated whether ionotropic glutamate receptors help to explain differential sensitivity in the locomotor-stimulant effects of low dose cocaine, with particular emphasis on the LCR/HCR rat model. We found that in LCRs, relative to HCRs, pGluN2B^Tyr-1472^ levels in the dSTR were 40% higher at a time after acute cocaine treatment (40 min) when locomotor activation differs markedly between LCRs and HCRs [27]. Further, pGluN2B^Tyr-1472^ levels had an overall inverse relationship with cocaine-induced locomotor activity. As expected, 7-day repeated cocaine treatment resulted in expression of cocaine-induced locomotor sensitization in LCRs, but not HCRs, so that the cocaine-induced locomotor activity was similar in all rats. These results are consistent with our past observations [11], [13], [15], [16]. Several other groups have explored cocaine sensitization and AMPAR changes after withdrawal of 1 day and 1 week or more from higher doses of repeated cocaine [19], [20], [21], [25]. Our study differs in that we measured 40 min after either an acute or repeated injection and used a lower dose of cocaine (10 mg/kg, i.p.) in our unique LCR/HCR model. We found that this repeated cocaine treatment increased total and cell surface GluA1, as well as increased GluA1^Ser-845^ phosphorylation, in the dSTR of the cocaine-treated group as a whole.

The differences in dSTR of pGluN2B^Tyr-1472^ that we observed after acute cocaine could help to explain the more ready development of sensitization in LCRs, as compared to HCRs. In support of this idea, selective antagonism of GluN2B has been found to prevent the development of cocaine-induced locomotor sensitization in mice [22] and rats [20], [28]. At glutamatergic synapses, pGluN2B^Tyr-1472^ has been shown to stabilize plasma membrane levels of NMDARs and prevent endocytosis [29]. Although we did not detect increased cell surface levels of GluN2B 40 min after acute cocaine, phosphorylation of pGluN2B^Tyr-1472^ also promotes lateral diffusion of GluN2B-containing NMDARs from extrasynaptic to synaptic sites [30]. This could result in increased pGluN2B^Tyr-1472^ at the synapse in the absence of any change in total or cell surface GluN2B subunit expression. The greater striatal NMDAR pGluN2B^Tyr-1472^ observed in LCRs after acute cocaine may enhance the sensitivity of their post-synaptic neurons to glutamate, and thereby contribute to their more ready development of locomotor sensitization, as compared to HCRs. It will be interesting in the future to determine if selective GluN2B inhibition prevents the preferential development of locomotor sensitization and conditioned place preference, and/or the greater motivation to self-administer cocaine in LCRs [13], [15].

Phosphorylation of GluA1 at Ser-845 has been shown to increase channel open time probability, drive AMPARs to the cell surface, and decrease GluA1 endocytosis [31]–[33]. We observed that repeated cocaine increased total and cell surface GluA1 and pGluA1^Ser-845^ in the dSTR of the cocaine group as a whole. These increases would enhance the response of postsynaptic receptors to glutamate and may relate to recruitment of more dorsal striatal circuits and maintenance of the marked cocaine-induced locomotor activation observed in all of the rats after repeated cocaine.

Kim et al. [34], found increased striatal GluA1 cell surface expression 24 hr after repeated cocaine treatment in juvenile mice. Prevention of this greater cell surface GluA1 expression suppressed the locomotor sensitization that developed in all of the mice after repeated cocaine, suggesting a relationship between AMPA receptor plasticity and enhanced locomotor activity in the dSTR. Drug-induced sensitization is a manifestation of neural plasticity, and an association between locomotor sensitization and drug seeking behavior has been noted [35], [36]. Altered ionotropic glutamate receptor signaling is essential for neural plasticity associated with the development of drug craving and seeking [37], [38], and it has been proposed that AMPAR cell surface changes may be involved in drug seeking behavior after withdrawal from self-administration [39], [40].

The LCR/HCR differences we found in NMDAR pGluN2B^Tyr-1472^ in the dSTR could be in response to acute cocaine, but it is also possible that they are innate. At this time we are unable to distinguish, and thus classify, rats as LCRs or HCRs without first injecting them with a single dose of cocaine. Nevertheless, the differences observed after acute cocaine in NMDAR pGluN2B^Tyr-1472^ in the dSTR were associated with the development of sensitization in LCRs.

While we found differences in the dSTR after cocaine treatment, much of the work on sensitization and glutamate receptor plasticity has focused on the NAc. The NAc has been shown to be important for the expression of behavioral sensitization [41] and for drug seeking behaviors [42]. However, it has also been suggested that, over time, the dSTR becomes important for habituated responses to cocaine and the rigid behaviors associated with drug seeking after prolonged self-administration [43]. Thus, the changes in total GluA1 and pGluA1^Ser-845^ observed in the dSTR here could be important for the development of incentive salience to cocaine and the drug craving that evolves over time.

In addition, although we found no significant differences in the NAc, it has recently been shown in rats that NAc cell surface GluA1 increases at 24 hr, but not at 30 min or 2 hr after an acute 15 mg/kg dose of cocaine [44]. Other repeated cocaine treatment studies, also using higher doses and longer withdrawal times, found that NAc cell surface GluA1 increases after withdrawal of a week or longer [21], [25], [45]. We focused on a relatively low dose of cocaine (10 mg/kg) and short times of withdrawal (40 min) because these are the conditions under which the LCRs and HCRs differ behaviorally. Further studies could help to determine if LCR/HCR cell surface differences would also be revealed in NAc or dSTR at 24 hr after acute cocaine, as well as after withdrawal of a week or more.

In addition to changes in AMPAR cell surface expression, repeated cocaine exposure produces substantial changes in glutamate neurotransmission both during withdrawal and subsequent re-exposure [46]. N-Acetyl Cysteine, a cysteine pro-drug used to normalize glutamate signaling in corticostriatal pathways, has been shown to reduce cocaine seeking in rats and craving in cocaine-dependent humans [37]. Further studies are necessary to ascertain if there are cocaine-induced LCR/HCR differences in extracellular glutamate and whether such changes may also contribute to the increased susceptibility of LCRs to the sensitizing, rewarding and reinforcing properties of cocaine.

In summary, the increased dSTR pGluN2B^Tyr-1472^ after acute cocaine in LCRs, but not HCRs, is consistent with the preferential development of locomotor sensitization in LCRs, as compared to HCRs. Repeated cocaine-induced increases in both total and cell surface GluA1 and pGluA1^Ser-845^ in dSTR may be important for the marked locomotor activation of all rats after repeated cocaine and could play a role in the incentive salience and drug seeking that develop over time. Our results further suggest that striatal NMDARs may contribute to the greater cocaine-induced behavioral plasticity observed in rats that are initially less activated by low dose cocaine. As we have found sensitization in our rat model is stable for at least a week [11], it will be interesting to examine whether there are glutamate receptor differences between LCRs and HCRs after long-term withdrawal.
